# Serratiopeptidase Niosomal Gel with Potential in Topical Delivery

**DOI:** 10.1155/2014/382959

**Published:** 2014-03-20

**Authors:** Ujwala A. Shinde, Shivkumar S. Kanojiya

**Affiliations:** Department of Pharmaceutics, Bombay College of Pharmacy, Kalina, Santacruz (East), Mumbai 400098, India

## Abstract

The objective of present study was to develop nonionic surfactant vesicles of proteolytic enzyme serratiopeptidase (SRP) by adapting reverse phase evaporation (REV) technique and to evaluate the viability of SRP niosomal gel in treating the topical inflammation. The feasibility of SRP niosomes by REV method using Span 40 and cholesterol has been successfully demonstrated in this investigation. The entrapment efficiency was found to be influenced by the molar ratio of Span 40 : cholesterol and concentration of SRP in noisome. The developed niosomes were characterized for morphology, particle size, and *in vitro* release. Niosomal gel was prepared by dispersing xanthan gum into optimized batch of SRP niosomes. *Ex vivo* permeation and *in vivo* anti-inflammatory efficacy of gel formulation were evaluated topically. SRP niosomes obtained were round in nanosize range. At Span 40 : cholesterol molar ratio 1 : 1 entrapment efficiency was maximum, that is, 54.82% ± 2.08, and showed consistent release pattern. Furthermore *ex vivo* skin permeation revealed that there was fourfold increase in a steady state flux when SRP was formulated in niosomes and a significant increase in the permeation of SRP, from SRP niosomal gel containing permeation enhancer. *In vivo* efficacy studies indicated that SRP niosomal gel had a comparable topical anti-inflammatory activity to that of dicolfenac gel.

## 1. Introduction

Recent advancements in biotechnology and genetic research have led to an increased surge of interest in the use of peptide and protein drugs. However, many of them require special formulation technologies to overcome drug-associated problems such as chemical and physical instability and poor bioavailability.

Proteolytic enzymes represent an important class of proteins and peptides with primary pharmacological use as anti-inflammatory and digestive agents. Among this category, serratiopeptidase (SRP) offers a powerful treatment for pain and inflammation with widespread use in arthritis, fibrocystic breast disease, chronic bronchitis, and carpal tunnel syndrome. SRP, an extracellular metalloprotease, is derived from the nonpathogenic enterobacteria* Serratia E15*. SRP comprises a polypeptide chain of 470 residues and one catalytic zinc ion per molecule with molecular weight 52 kDa. SRP is given orally at a dose of 5–10 mg three times a day. Formulations of SRP are available mainly in the form of enteric coated tablet (Danzen, Takeda Japan). The oral bioavailability of these peptide drugs is generally very low, owing to the acidic conditions of the stomach, proteolytic activity of gastrointestinal tract, and poor permeability across intestinal mucosa. In order to increase the stability of SRP (reduction in acid hydrolysis) and hence to improve bioavailability, various other approaches of delivering the enzymes at the target site have been reported, which include enzyme-entrapped Eudragit S100 microspheres [1], liposomal formulations of serratiopeptidase [2, 3], alginate gel—encapsulated with serratiopeptidase, chitosan-coated ceramic nanocores containing serratiopeptidase [4],* in situ* cubic phase transforming system of glyceryl monooleate containing serratiopeptidase [5], tetracycline-serratiopeptidase-containing periodontal gel [6], and polar lipid-based lipospheres [7].

Orally administered SRP has been reported to show systemic side effects like anticoagulant effects. Therefore, to reduce systemic side effects and to increase local effects one of the approaches is to deliver SRP by topical route [8]. The application of niosomal drug delivery for dermal drugs is one of the numerous strategies for effective modulation of drug release and penetration enhancement. In recent years many researchers substantiating that the vesicular structures, such as liposomes, niosomes, ethosomes, and transfersomes, are acting as the best carrier for administration of drugs across the skin. These vesicular structures act as carriers for drugs and help to overcome the barrier properties of the skin. Niosomes, a vesicular drug delivery system, has been used as a drug carrier or reservoir due to its intrinsic skin penetration enhancing properties and more stability than other vesicular systems like liposomes, ethosomes, and so forth. Niosomes are globular submicroscopic structures and are prepared using nonionic surfactants such as Tweens and Spans [9]. The present study is based on the hypothesis that incorporation of SRP into niosomes will improve its penetration across the skin, which will in turn improve local anti-inflammatory efficacy of SRP upon topical administration.

## 2. Materials and Methods

### 2.1. Materials

Serratiopeptidase (SRP) was obtained as a gift sample from Advanced Enzyme Technology, Thane, India. Sorbitan monopalmitate (Span 40) was a gift sample from Nikko Chemicals, Japan. Cholesterol was purchased from Loba Chemicals Ltd, India. Other materials and solvents used were of analytical grade.

### 2.2. Preparation of SRP Niosomes

Niosomes were prepared by REV method as described by Guinedi et al. [10]. SRP niosomes of 5 mL batch size were prepared. Briefly, 1 mL solution of 0.1 M Span 40 was transferred in 100 mL round bottom flask containing 10 mL mixture of ether : chloroform (1 : 1). Cholesterol solution (0.1 M), 1 mL, was added in the above mixture such that Span 40 and cholesterol were in 1 : 1 molar ratio. Tris-buffer, 1 mL, containing 10 mg SRP was added such that organic to aqueous phase ratio was 10 : 1. The mixture was then sonicated for 5 minutes in an ultrasonicator water bath (Expo, India). Liquid emulsion so formed was dried to a semisolid gel in a rotary evaporator (Superfit, Mumbai) at 40 ± 2°C (above the gel-to-liquid-crystalline phase transition temperature of the Span 40) by applying vacuum (400–600 mmHg). Resultant viscous dispersion was finally diluted further with Tris-buffer (pH 7.0) to 5 mL. Niosomal dispersion was sonicated for up to 2 hrs with intermittent sonication (30 second) in a bath sonicator at room temperature after every one hour to reduce the vesicle size. Unentrapped drug was removed by centrifugation method as discussed by Shahiwala and Misra [11] and drug (SRP) loaded niosomes were stored at 2–8°C for further characterization. SRP niosomes were prepared by varying Span 40 : cholesterol molar ratios from 1 : 0.5 to 1 : 2.5 and drug loading 5–15 mg and their effect of entrapment efficiency was investigated.

### 2.3. Determination of Entrapment Efficiency of SRP Niosomes

The entrapment efficiency of SRP in niosomes was determined indirectly. Niosomal dispersion, 1 mL, was transferred to eppendorf tube of 1.5 mL capacity using a 1 mL micropipette (Tarsons, India) and centrifuged at 11,000 rpm for 30 minutes using a refrigerated centrifuge at 4°C (Remi C-30 BL, India). The collected supernatant, 0.5 mL, was appropriately diluted with Tris-buffer pH 7.0 and analyzed by proteolytic assay method for liberated L-tyrosine content [12]. Using the following formula percent entrapment efficiency of SRP in niosomes was calculated:

(1) Percentage  entrapment  (%E)  =[Entrapped  drug  (mg)Total  drug  added  (mg)]×100.

### 2.4. Characterization of SRP Niosomes

#### 2.4.1. Particle Size Distribution and Polydispersity Index

The particle size distribution of niosomes was evaluated by photon correlation spectroscopy (PCS) on a Beckman coulter N5 submicron particle size analyzer (coulter corporation, USA).

#### 2.4.2. Surface Morphology by Transmission Electron Microscopy (TEM)

The sample of niosomes (5–10 *μ*L) was dropped onto Formvar coated copper grids Philips CM-200 (Philips, Netherland). After complete drying, sample was stained using 2% (w/v) phosphotungstic acid. Digital Micrograph and Soft Imaging viewer software were used to perform the image capture and analysis, including particle sizing. The stained grid was air-dried and observed. Image was visualized on screen under the electron microscope (PHILIPS-CM 200), SAIF, IIT Bombay, Mumbai, and photographed.

#### 2.4.3. *In Vitro* Release Studies

*In vitro *drug release profiles of niosomal dispersion weredetermined in Tris-buffer pH 7.0, using cellulose acetate membrane (0.45 *μ*, Sartorious Ltd, Germany) at 37°C under magnetic stirring. Cellulose acetate membrane paper was soaked overnight in Tris-buffer pH 7.0 to open the membrane chains and dried to room temperature to remove the entire medium. Paper was mounted on Franz diffusion cell equipped with 16.5 mL of Tris-buffer in receptor compartment. Temperature was maintained at 37°C. SRP niosomes equivalent to 5 mg of SRP were placed on the donor side of the paper. Receptor fluid was constantly stirred with a small bar magnet. At predetermined time intervals (i.e., 0, 15, 30, 45, 60, 120, 180, 240, 300, and 360 min), 1 mL of sample aliquot was withdrawn and replaced with equal amount of fresh Tris-buffer. Each withdrawn aliquot was analyzed for SRP content by proteolytic method and percent cumulative release was calculated.

### 2.5. Preparation of SRP Niosomal Gel

Optimized SRP niosomal formulation was gelled by dispersing xanthan gum (2% w/w). Briefly, SRP niosome pellet quantity equivalent to 1% w/w SRP was weighed and transferred to a 50 mL beaker containing 5 mL of distilled water. Mixture was stirred to obtain homogenous dispersion. Gelling agent, xanthan gum, was weighed accurately and added with stirring to niosomal dispersion. Weight of mixture was adjusted upto 10 gm by adding drop by drop distilled water. After completion of addition the mixture was allowed to hydrate to its maximum capacity by keeping it at room temperature for 2 hrs.

### 2.6. Physicochemical Evaluation of the SRP Niosome Based Gel

The topical niosomal gels were evaluated for particle size, drug content, spreadability, pH, and viscosity. Particle size of SRP niosomal gel was determined by diluting, 1 g of gel with 250 mL of doubled distilled water. The dispersions were observed visually; and particle size was determined in triplicate immediately on a Beckman Coulter N5 submicron particle size analyzer (Coulter Corporation, U.S.A.). The drug content was quantified by dissolving 1 gm gel in Tris-buffer (pH 7.0); volume was made to 10 mL in volumetric flask; it was diluted appropriately and analyzed for SRP content using proteolytic assay. The spreadability of the gel was determined as per technique described by Bachhav and Patravale [13]. Briefly, 0.5 g gel was placed within a circle of 1 cm diameter premarked on a glass plate over which a second glass plate was placed. A weight of 500 g was allowed to rest on the upper glass plate for 5 min. The increase in the diameter due to the spreading of the gels was noted. The pH niosomal gels were measured using the digital pH meter (Universal Enterprises) at room temperature. The pH meter was standardized using pH 4.0 and 7.0 standard buffers before use. Rheological measurements were performed on cone and plate Brookfield Viscometer (Cap 2000) using spindle number 4. Viscosity parameters were collected at different rpm (100 rpm to 500 rpm) with 1-minute equilibration time at every rpm. Samples (0.5 gm) were applied to the lower plate using a spatula to ensure that formulation shearing did not occur. The measurements were performed at 25°C in triplicate. Rheogram was constructed by plotting the viscosity versus rpm.

### 2.7. *Ex Vivo* Skin Permeation Study

The* ex vivo* skin permeation of SRP niosomal dispersion and SRP niosomal gel was performed with Franz diffusion cell using abdominal skin of male Wistar rat. The abdominal skin of rat was excised and the adhering fat and other visceral tissues were removed. The skin membranes were first hydrated for 30 minutes in the Tris-buffer solution (pH 7.0) at room temperature (30°C) to remove the extraneous debris and leachable enzymes. They were then placed between the donor and receptor compartments of the cells with the dermal side in direct contact with the receptor medium. Approximately 16.5 mL Tris-buffer (pH 7.0) was placed in the receptor compartment. Its temperature was maintained at 37 ± 0.5°C using a thermostatic water bath, and it was stirred at 600 rpm throughout the experiment. Donor compartment contained formulation equivalent to 5 mg of SRP. Aliquots (1 mL) were withdrawn at predetermined time intervals and then immediately analyzed by the proteolytic assay method. The data was analyzed to assess various permeability parameters [14].

#### 2.7.1. Steady-State Flux

Flux is defined as the rate of diffusion or transport of a substance across a permeable membrane. After drug permeation reached steady state, the steady-state flux was calculated using the following equation:

(2) $$\begin{array}{c} \text{Steady}\;\;\text{state}\;\;\text{flux}\;\;\left(\text{Jss}\right)=\frac{dM}{S}\cdot dt, \end{array}$$

where *dM* is the amount of drug that permeates through a unit cross section area and *S* is per unit time, *t*. The slope of the steady-state portion of the permeation curve created by plotting the cumulative amount of drug, permeated in micrograms versus time in hours, is the flux.

#### 2.7.2. Permeability Coefficient

The permeability coefficient through the membrane (*Kp*) was determined according to the following equation:

(3) $$\begin{array}{c} \text{Permeability}\;\text{coefficient}\;\;\left(Kp\right)=\frac{\left(\text{Jss}\cdot H\right)}{C_{0}}, \end{array}$$

where *H* is the thickness of membrane and *C*_0_ is the initial drug concentration.

#### 2.7.3. Enhancement Ratio

This factor was calculated to find the relative enhancement in the flux of formulations with respect to the reference enhancement ratio. The enhancement ratio was estimated according to the following equation:

(4) $$\begin{array}{c} \text{Enhancement}\;\;\text{ratio}\;\;\left(\text{Er}\right)=\frac{\text{Jss}\;\;\text{formulation}}{\text{Jss}\;\;\text{reference}}. \end{array}$$

### 2.8. Physical Stability of SRP Niosomes and Niosomal Gel

Physical stability studies were carried out to investigate the leaching of drug from niosomes and gel formulation during storage. Optimized SRP niosomes and gel formulation, composed of Span 40 and cholesterol in a 1 : 1 molar ratio with 10 mg SRP, were sealed in 20 mL glass vial and stored at refrigeration temperature (2–8°C) for a period of 3 months. Samples from each batch were withdrawn at definite time intervals; the residual amount of the drug in the vesicles was determined by proteolytic assay.

### 2.9. *In Vivo* Efficacy Evaluation

#### 2.9.1. *In Vivo* Evaluation of Anti-Inflammatory Activity of SRP Niosomal Gel

The relative anti-inflammatory efficacy of the tested preparations and the commercially available reference preparation were measured and compared [10, 15]. Male Wistar rats weighing 160–180 g were used in this experiment. All the experimental procedures and protocols used in this study were reviewed and approved by the Institutional Animal Ethical Committee (IAEC), constituted as per the requirement of Committee for the Purpose of Control and Supervision of Experiments on Animals before the study was started.

The anti-inflammatory action was evaluated using the carrageenan-induced hind paw edema method with a slight modification. Rats were randomly selected and divided into four groups of six animals each. These groups were divided, according to the formulae administered, into control (vehicle base), plain SRP gel, niosomal SRP gel, and reference gel (Diclofenac gel) groups. The animals were housed in polypropylene cages at 25 ± 1°C and 60 ± 5% relative humidity, with free access to food and water. One day prior to application of the trial formulation, the hair on the dorsal surface of the rat was shaved. Plain SRP gel, niosomal SRP gel, reference gel, and control formulae were applied on the shaved dorsal surface by gentle rubbing for 15 seconds. After five hours, 0.1 mL of 1% w/v suspension of carrageenan in normal saline was injected into the subplantar region of the right hind paw of all control and treated rats. Edema volume, in terms of thickness, was measured in all four groups at hours 2, 4, and 6 after carrageenan injection using a micrometer (Ozaki Ltd, Tokyo, Japan). The induced thickness was measured by placing the foot of the rat between the anvil and spindle of the micrometer. Mathematically, the degree of swelling can be expressed as

(5) $$\begin{array}{c} \%\;\text{change}\;\text{in}\;\text{hind}\;\text{paw}\;\text{thickness}=\frac{\left(C_{t}-C_{0}\right)}{C_{0}}\times 100, \end{array}$$

where *C*_*t*_ is hind paw thickness at hours 2, 4, and 6 after injection of carrageenan and *C*_0_ is the initial hind paw thickness before injection of carrageenan.

#### 2.9.2. Statistical Analysis

Statistical analyses of the anti-inflammatory effects of the SRP niosomal gel formulation were performed using one-way analysis of variance (ANOVA) followed by Tukey test. Results are expressed as a mean ± standard deviation. A statistically significant difference was accepted at *P* < 0.05.

## 3. Results and Discussion

### 3.1. Entrapment Efficiency

Results of preliminary studies revealed that the stable niosomes of SRP could be prepared using cholesterol and Span 40 by adopting REV technique. The entrapment efficiency is the crucial parameter in the preparation niosomal formulation and formulation scientist's efforts are always directed towards achieving high entrapment efficiency. The concentration of the surfactant and the concentration of cholesterol are the main factors in niosomes that govern the encapsulation efficiency and particle size of the niosomal vesicles. Hence, the effects of these factors on encapsulation efficiency were investigated to optimize the SRP niosomes.

#### 3.1.1. Effect of Cholesterol Content

The SRP niosomes were prepared by changing the molar ratio of surfactant to cholesterol to examine the effect of different amounts of cholesterol entrapment efficiency. Cholesterol is one of the common additives included in the formulation in order to prepare stable niosomes. Cholesterol is known to abolish the gel to liquid phase transition of niosome systems, which could effectively prevent the leakage of the drug from niosomes [16].  Table 1 revealed the change in cholesterol and Span 40 concentration on entrapment efficiency of SRP niosomes. Encapsulation efficiency of niosomal dispersions was in the range of 45–55%. The quantity of SRP entrapped was increased with increasing cholesterol content. Formulation of the niosome with molar ratio of 1 : 1 was most beneficial for the efficient encapsulation, and excess cholesterol was unfavourable. According to Finean excessive concentration of cholesterol could result in cluster formation leading to nonuniform distribution of drug along the bilayers affecting the integrity of the membrane [17]. In the study published by Hao et al. the authors reported similar trend for colchicines niosomes which is water soluble [18]. It implied that equal molarity of Span 40 and cholesterol makes the membrane compact and well organized.

#### 3.1.2. Effect of Surfactant Concentration

Influence of levels of Span 40 was examined by altering its total concentration, while keeping the concentration of cholesterol invariable. The experiments demonstrated that Span 40 concentration had an impact on entrapment.  Table 1 shows that the increase in concentration of Span 40 increases the entrapment efficiency of SRP. Maximum entrapment efficiency 54.82 ± 2.08 was achieved at molar ratio 1 : 1 of Span 40 and cholesterol. Low drug entrapment at low Span 40 concentration could be attributed to the small number of niosomes produced by dilute surfactant solution. These finding were concordant with the results reported by Hao et al. for colchicine which is water soluble [18].

#### 3.1.3. Effect of SRP Loading

The influence of drug concentration on encapsulation was examined by varying the amount of SRP added while keeping the total concentration of surfactant lipid at 1 : 1 molar ratio and results are reported in Table 2. It was seen that niosomes prepared in this study showed a good encapsulation capacity. It was found that at lower drug level the entrapment was low. With increase in drug loading the entrapment was increased; however, beyond optimum drug loading there was no significant change in the percent drug entrapment but particle size was significantly increased. The maximum percentage drug entrapment, that is, 54.01% ± 3.50, was observed at 10 mg SRP loading with minimum vesicle size (362 ± 2.20) and PI (0.28). Thus, formulation containing 10 mg SRP in Span 40 : cholesterol in 1 : 1 molar ratio was gelled by adding xanthan gum.

### 3.2. Characterization of SRP Niosomes

#### 3.2.1. Vesicle Size

Vesicle size of SRP niosomal suspensions is reported in Table 1. All of the vesicles formed were in the size ranging from 325 ± 4.01 nm to 988 ± 2.21 nm. Results in Table 1 revealed that molar ratio of Span 40 and cholesterol has a significant effect on size of niosomal vesicles. From the observations, it was evident that changes in the concentration of cholesterol significantly affect the particle size. The vesicle size was increased with increase in cholesterol concentration. This may be due to the rigid structure of cholesterol. Results of polydispersity index (0.25–0.35) revealed that the niosomal vesicles were uniformly distributed and homogenous mixture.

#### 3.2.2. Surface Morphology by Transmission Electron Microscopy (TEM)

**Figure 1:**
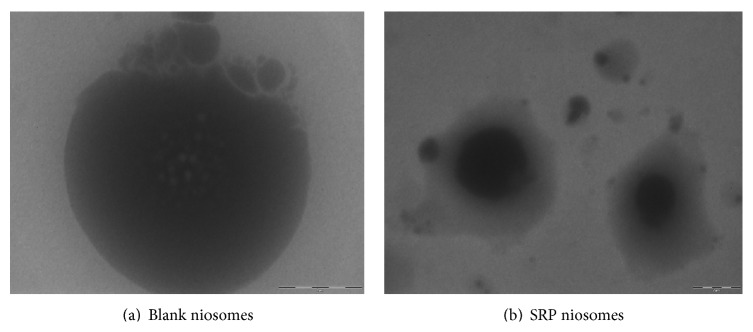
Transmission electron photomicrographs of SRP niosomes.

TEM is widely used to study image structures near to the atomic level and has been used in imaging morphology of the niosomes. In the present study, TEM study was carried out to find out the size and shape of niosomes. The TEM photomicrograph of empty and SRP loaded niosomes is shown in Figures 1(a) and 1(b), respectively. The drug loaded niosomes appeared as dark spheres with faint outlines. However, empty niosomes without drug appeared dark against a brighter background showing inner bright spherical core and surrounded by spherical dark background.

#### 3.2.3. *In Vitro *Release of SRP from Niosomes

**Figure 2:**
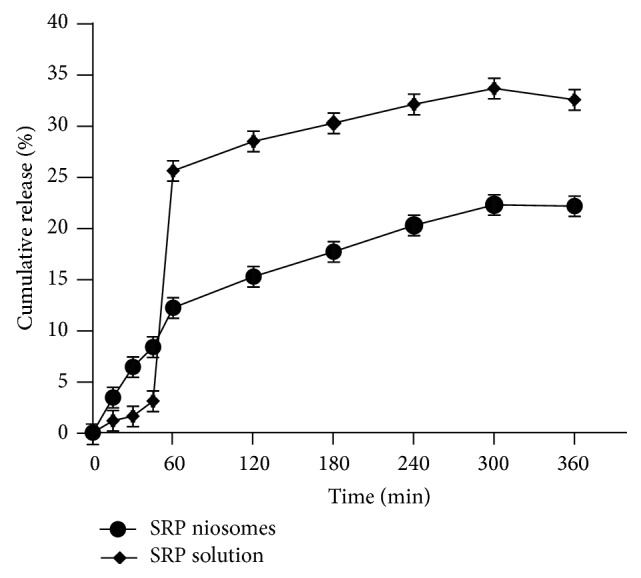
*In vitro* release of SRP through cellulose acetate membrane.

*In vitro* release studies were carried out in triplicate using cellulose acetate membrane (0.45 *μ*) to assess the release of SRP from SRP niosomal dispersion. Percent cumulative release of SRP from SRP niosomal dispersion is indicated in Figure 2. The percent cumulative release from SRP niosomal dispersion was found to be slow and sustained release as compared to percent cumulative release from aqueous solution of SRP. At the end of sixth hour the drug release from noisome was 22.01 ± 0.79%, whereas from aqueous solution of SRP it was 32.47 ± 0.65%. Hence, sustained release pattern was observed. It is clear that the release is slower from multilamellar niosomes. This may be attributed to the fact that multilamellar vesicles consist of several concentric spheres of surfactant which are rigidized by cholesterol. Therefore, the diffusion of SRP entrapped in the multilamellar vesicles would be expected to occur over a prolonged period of time. Cholesterol is known to abolish the gel to sol phase transition of niosome systems; hence resulting niosomes are less leaky thus reducing SRP release from niosomes.

### 3.3. Physicochemical Characterization of SRP Gel

**Figure 3:**
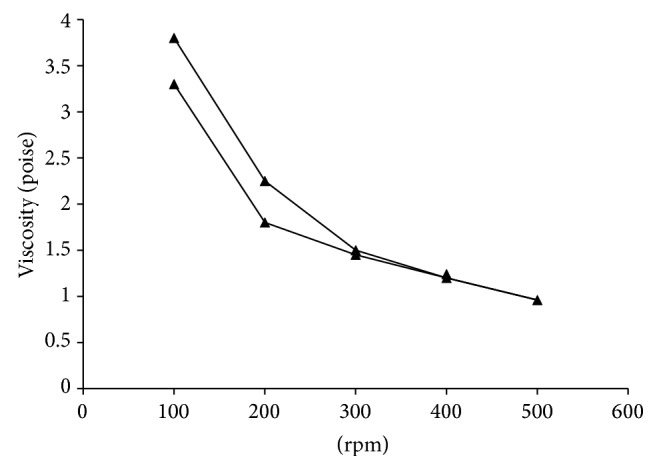
Rheology of SRP niosomal gel.

It was noted that there was change in particle size when niosomes were formulated in gel form using xanthan gum. In niosomal gel the particle size was increased from 362.21 *μ* to 978.8 *μ*. Similar results were reported by Antunes et al. for diclofenac sodium niosomal Pluronic gel. The increase in particle in SRP niosomal gel could be due to the formation of polymer layer along the vesicle surface [19]. The low values of polydispersity index indicated that there was no aggregation of vesicles in the gel. The drug content of the SRP niosomal gels is as shown in Table 3 and was well within limits. There was no degradation of SRP in the niosomes when formulated as niosomal gels. The larger the area the better the spreadability and good ability to spread over larger surface area. Formulation gelled with xanthan gum showed better spreadability (area 31.15 ± 0.15 cm^2^), whereas the area for marketed diclofenac gel formulation was found to be 18.08 ± 0.28 cm^2^, indicating that the spreadability of SRP niosomal gels was better than that of marketed diclofenac gel. This could be because of the loose gel matrix nature of niosomal gel due to presence of vesicles. The pH range for skin is 6–8. As shown in Table 3, the pH values of all formulations were found to be compatible for topical application. From the rheogram curve (Figure 3) we could conclude that SRP niosomal gel formulation exhibited pseudoplastic flow behavior. The pseudoplastic (shear thinning) behavior of xanthan gum gel has been observed previously [13]. SRP niosomal gel had viscosity in the range of 3.8 poise to 3.3 poise.

### 3.4. *Ex Vivo* Permeation Studies

**Figure 4:**
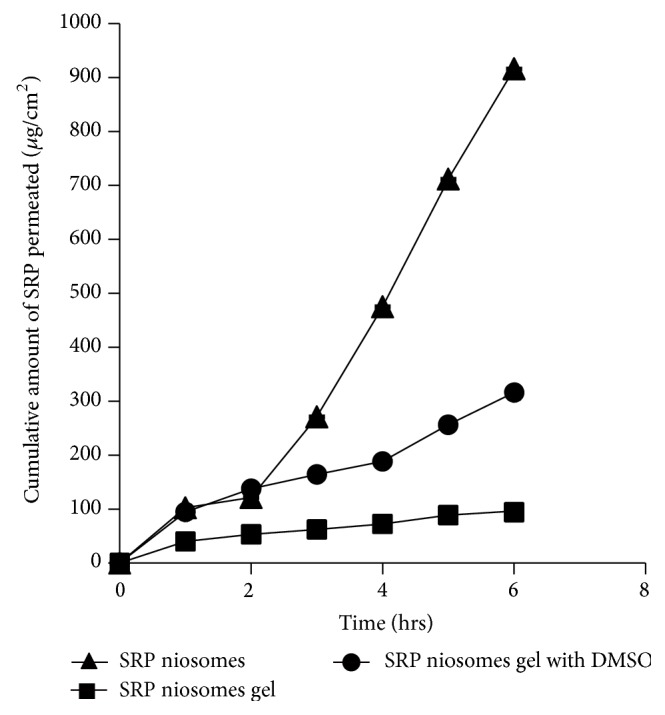
Cumulative amount of SRP permeated through rat skin.

Niosomes are composed of nonionic surfactants, which are biocompatible and relatively nontoxic and themselves serve as excellent penetration enhancers [20]. Niosome formulation is expected to penetrate the stratum corneum and exist intact in the whole horny layer. Once it enters into the stratum corneum, niosomes may simultaneously alter both the lipid and the polar pathways. To evaluate skin penetration of SRP,* ex vivo* skin penetration studies were performed using Wistar rat skin. In this study the permeation data obtained from SRP niosomes and niosomal gel was compared with aqueous solution of SRP.  Table 4 and Figure 4 show skin permeation of SRP niosomes and niosomal gel. The cumulative amount of SRP permeated from SRP aqueous solution was very low (i.e., 189.18 ± 1.27 *μ*g/cm^2^) as compared to niosomal dispersion of SRP (i.e., 916.22 ± 0.64 *μ*g/cm^2^); it could be because of high molecular weight of SRP, high water solubility, and low permeability. The mean flux of SRP from niosomal SRP dispersion was high (116.7 ± 2.15 *μ*g/cm^2^ hr) with high permeability coefficient (0.306). The high mean flux (116.7 ± 2.15 *μ*g/cm^2^ hr) in niosomal dispersion could be due to penetration enhancement effect of nonionic surfactants in vesicles. However, cumulative amount permeated through niosomal gel was low, that is, 96.45 ± 1.25 *μ*g/cm^2^. SRP is a hydrophilic protein (proteolytic) enzyme and has high molecular weight of about 52 kDa; thereby, its skin permeation is poor and therefore permeation through skin becomes rate limiting step. Thus, in the case of peptides the permeation enhancement required is substantially greater due to their hydrophilicity and high molecular weight. Thus, to enhance SRP permeation through the rat skin combined strategies of niosomal gels formulated with penetration enhancers were studied. The study performed by Foldvari et al. showed that encapsulation of interferon (INF)-*α* in liposomes leads to increased deposition in the skin, but increased penetration of macromolecules through the skin has not been demonstrated [21]. Several penetration enhancement techniques such as chemical modification to form a conjugate with increased lipophilicity and encapsulation into hydrophobic carriers and incorporation of penetration enhancers which chemically or physically reduce the stratum corneum barrier have been developed to overcome the skin barrier and to facilitate the permeation of such high molecular peptides and proteins through the skin. Thus, to enhance SRP permeation through the rat skin combined strategies of niosomal gels formulated with penetration enhancers were studied. To evaluate the combined effect of the enhancers with non-onic surfactant vesicles on skin penetration of SRP, that is, to improve the* ex vivo* skin permeation, the SRP niosomal gels were prepared by incorporating 50% dimethylsulfoxide (DMSO). SRP niosomal xanthan gum gels with 50% DMSO showed 316.43 ± 1.5% cumulative release. In presence of penetration enhancer the values of the mean flux, permeability coefficient, and enhancement ratio were increased.

### 3.5. Physical Stability

**Figure 5:**
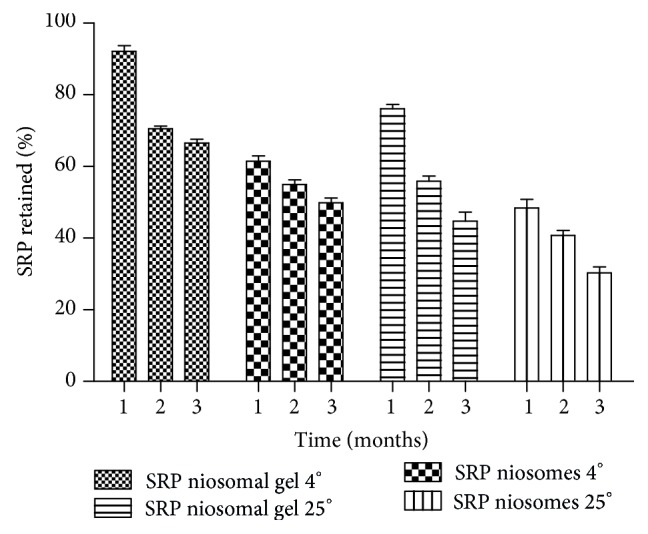
Physical stability of SRP in niosomes and niosomal gel.

Physical stability was performed in order to investigate the niosome's ability to retain entrapped drug during a defined period of time. The percentages of SRP retained after period of 3 months in niosomes were 49.91 ± 1.29 and 30.31 ± 1.62 and niosomal gel formulation 66.51 ± 1.15 and 44.67 ± 2.5 at refrigerated temperature and 25°C, respectively (Figure 5). The studies also indicate approximately 61.43 ± 1.5% and 92.16 ± 1.54 of SRP were retained in niosomal formulation and gel, respectively, for a period of 30 days at refrigerated conditions. The enzyme leakage at elevated temperature could be due to changes in rigidity of niosomal vesicles. At higher temperature there could be deformation of gel state of cholesterol. Similar results are reported by Agarwal and Katare for miconazole nitrate liposomes [22]. However, the rate of enzyme leakage was reduced when the niosomal formulation converted into gel upon addition of xanthan gum.

### 3.6. *In Vivo* Efficacy Studies

**Figure 6:**
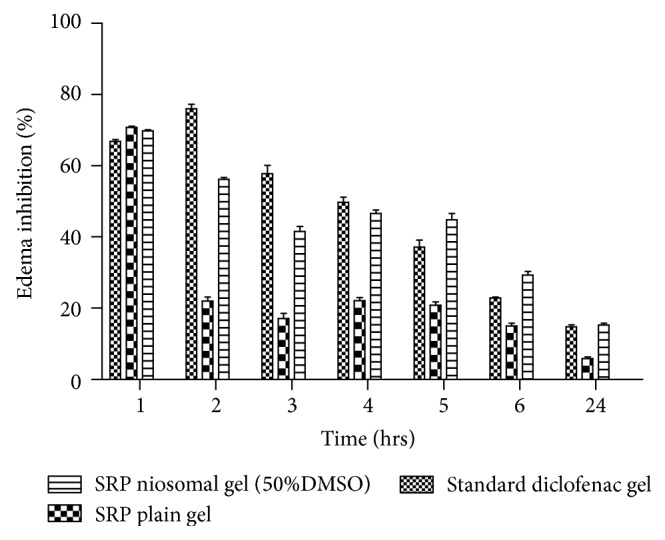
Anti-inflammatory efficacy.

The anti-inflammatory activity of the SRP can be evaluated on the basis of the ability of the administered drug to inhibit edema produced in the rat hind paw after injection of carrageenan [15]. Comparison of the increase in paw volume which was produced after challenge with the phlogistic agent in untreated rats and drug treated rats can be used to evaluate the anti-inflammatory activity of drugs. The plot of percent inhibition of edema V/s time after challenge was studied to detect any enhancement in anti-inflammatory activity of the drug when given as a niosomal gel. The results of the rise in edema volume were calculated as percent edema and represented graphically in Figure 6.

Positive control group animals showed distinct formation of edema which was not reduced during the test significantly. Initially the standard group showed greater percent edema inhibition (76.01 ± 3.19% at 2 hours) and then the effect was reduced significantly with time up to 24 hrs. The reason for high initial percent edema inhibition at 2 hrs followed by rapid decline to 14.77 ± 1.94% could be due to small molecular size of diclofenac which was rapidly permeated, absorbed, and excreted from the body. The group treated with plain SRP gel showed that percent edema inhibition (22.05 ± 2.56 at 2 hr) was much less compared to the standard and SRP niosomal gel with 50% DMSO. Percent inhibition then gradually reduced to 5.68 ± 1.60% in 24 hrs. The values of percent edema inhibition were significantly lower at all the time points. This may be due to less penetration and absorbance of SRP from plain SRP gel because of its large molecular weight and absence of penetration enhancers in plain drug gel. In case of the niosomal gel containing 50% DMSO, by the second hour the reduction in percent inhibition (56.35 ± 1.28) was less as compared to standard group, which could be attributed to large molecular weight of SRP. However, the percent inhibition was gradually decreased to 15.19 ± 1.26% in case of niosomal gel containing 50% DMSO at the end of 24 hours. The high drug deposition and its slow diffusion from the skin with niosomal gel may have resulted in gradual increase in the percent edema inhibition between 3 and 5 hrs compared to standard. The significant higher skin retention of the niosomal SRP containing 50% DMSO resulted in higher partitioning of the SRP into the rat paw which may be responsible for its prolonged and enhanced anti-inflammatory activity. At a similar level of drug, the mean values of the percent inhibition produced by SRP niosomal gel containing 50% DMSO were higher at most of the time points as compared to those produced by plain SRP gel. The higher inhibition at 2 hr indicates faster onset of anti-inflammatory action from SRP niosomal gel with 50% DMSO compared to plain SRP gel.

**Figure 7:**
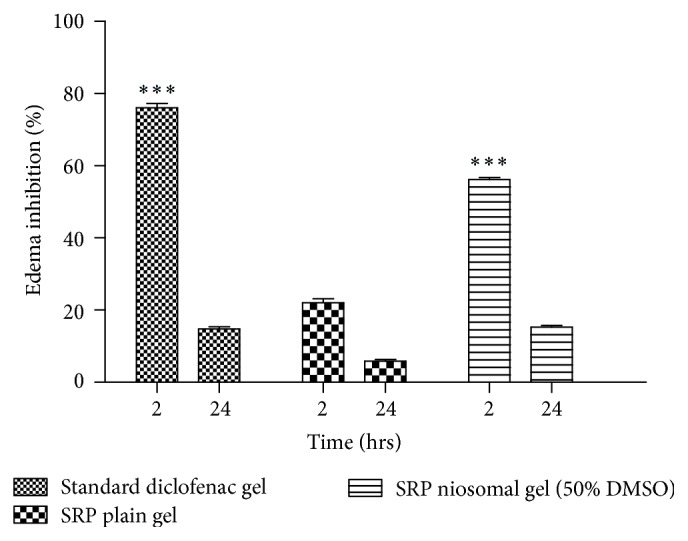
Anti-inflammatory efficacy of SRP niosomal gel at 2 h and 24 h. Data are the mean ± SEM (*n* = 6), one-way ANOVA. ^***^ *P* < 0.05 compared with control (Tukey test).

Mean percent inhibition and standard deviation were calculated for 2 h and 24 h data and are shown in Figure 7. Results are presented as mean ± SEM (*n* = 6). One-way ANOVA test followed by Tukey's test (^***^ *P* < 0.05). The anti-inflammatory activity of SRP noisome gel containing was significantly different from the control group. Results demonstrated that the anti-inflammatory effect of SRP niosomal gel and diclofenac gel was comparable. The niosomal gel formulation can provide consistent and prolonged anti-inflammatory effect and may help in improving therapeutic index of the formulation and is also expected to minimize the side effects due to selective buildup of drug concentration at the site of action.

## 4. Conclusion

Thus, foregoing results indicate that under optimized conditions serratiopeptidase can be successfully incorporated in niosomal system. Serratiopeptidase niosomes were successfully developed using Span 40 : cholesterol 1 : 1 molar ratio by adapting reverse phase evaporation technique. Xanthan gum could be effectively used as gelling agent for the preparation of serratiopeptidase noisome gel with DMSO as penetration enhancer.* In vitro* skin permeation, mean flux, and permeability coefficient of optimized formulation were found to be more than gel containing serratiopeptidase solution.* In vivo* studies indicated a promising application of serratiopeptidase niosomal gel as anti-inflammatory gel.

## Figures and Tables

**Table 1:** Effect of Span 40 : CH ratio on percent entrapment efficiency.

Sr. number	Span 40 : CH with 10 mg SRP loading	Percent drug entrapment	Particle size (nm)	Polydispersity index
1	1 : 0.5	47.98 ± 2.49	325 ± 4.01	0.28
2	1 : 0.7	51.78 ± 3.50	348 ± 3.55	0.41
3	1 : 1	54.82 ± 2.08	362 ± 2.21	0.28
4	1 : 1.5	49.85 ± 1.85	864 ± 4.64	0.35
5	1 : 2	49.25 ± 3.49	988 ± 2.21	0.61
6	0.5 : 1	45.78 ± 2.28	556 ± 1.04	0.45
7	0.7 : 1	48.97 ± 2.56	464 ± 3.45	0.25
8	1.5 : 1	54.55 ± 2.05	845 ± 5.79	0.48
9	2 : 1	50.94 ± 4.56	680 ± 4.21	0.54

**Table 2:** Influence of drug loading on percent entrapment efficiency in niosomes with Span 40 : cholesterol 1 : 1.

Concentration of SRP (mg)	Percent drug entrapment	Particle size (nm)	Polydispersity index
5	34.85 ± 2.61	369 ± 1.03	0.39
7	45.19 ± 3.12	386 ± 0.59	0.35
10	54.01 ± 3.50	362 ± 2.21	0.28
12	54.44 ± 5.15	898 ± 2.35	0.29
15	54.96 ± 4.80	754 ± 4.89	0.3

**Table 3:** Physicochemical characterization of SRP niosomal gel.

Parameters	Results
Particle size	978.80 ± 1.8
Polydispersity index	0.410
Drug content (% w/w)	99.26 ± 0.25
Spreadability (area in cm^2^)	31.15 ± 0.15
pH	6.71 ± 0.21
Marked diclofenac gel	18.08 ± 0.28

**Table 4:** Cumulative amount permeated, flux, and permeability coefficient of SRP across excised rat skin.

Formulation	Cumulative amount permeated (*μ*g/cm^2^)	Mean flux (*μ*g/cm^2^·hr)	Permeability coefficient (cm^2^/hr) × 10^3^	Enhancement ratio
SRP solution in Tris-buffer	189.18 ± 1.27	29.7 ± 0.41	59.40 ± 0.82	—
SRP niosomal dispersion	916.22 ± 0.64	68.79 ± 0.35	137.58 ± 0.7	2.32
SRP niosomal gel without DMSO	96.45 ± 1.25	4.12 ± 0.68	8.24 ± 1.37	0.14
SRP niosomal gel with DMSO	316.43 ± 1.5	20.43 ± 0.27	40.87 ± 0.53	0.69
